# Biomechanical study of a low-cost external fixator for diaphyseal fractures of long bones

**DOI:** 10.1186/s13018-020-01777-5

**Published:** 2020-07-06

**Authors:** Kouamé Jean-Eric Kouassi, Olivier Cartiaux, Loic Fonkoué, Christine Detrembleur, Olivier Cornu

**Affiliations:** 1 Experimental and Clinical Research Institute (IREC), Neuro-Musculo-Skeletal Pole (NMSK), Université Catholique de Louvain, Tour Pasteur +4 – 53 Avenue Emmanuel Mounier, 1200 Brussels, Belgium; 2 Department of Orthopedics and Trauma, Cliniques Universitaires Saint-Luc, Université Catholique de Louvain, Brussels, Belgium; 3 Department of Health Engineering, ECAM Brussels Engineering School, Haute Ecole “ICHEC-ECAM-ISFSC”, Brussels, Belgium

**Keywords:** Biomechanical testing, External fixators, Low-cost, Stiffness

## Abstract

**Background:**

External fixation improves open fracture management in emerging countries. However, sophisticated models are often expensive and unavailable. We assessed the biomechanical properties of a low-cost external fixation system in comparison with the Hoffmann® 3 system, as a reference.

**Methods:**

Transversal, oblique, and comminuted fractures were created in the diaphysis of tibia sawbones. Six external fixators were tested in three modes of loading—axial compression, medio-lateral (ML) bending, and torsion—in order to determine construction stiffness. The fixator construct implies two uniplanar (UUEF1, UUEF2) depending the pin-rods fixation system and two biplanar (UBEF1, UBEF2) designs based on different bar to bar connections. The designed low-cost fixators were compared to a Hoffmann® 3 fixator single rod (H3-SR) and double rod (H3-DR). Twenty-seven constructs were stabilized with UUEF1, UUEF2, and H3-SR (nine constructs each). Nine constructs were stabilized with UBEF1, UBEF2, and H3-DR (three constructs each).

**Results:**

UUEF2 was significantly stiffer than H3-SR (*p* < 0.001) in axial compression for oblique fractures and UUEF1 was significantly stiffer than H3-SR (*p* = 0.009) in ML bending for transversal fractures. Both UUEFs were significantly stiffer than H3-SR in axial compression and torsion (*p* < 0.05), and inferior to H3-SR in ML bending, for comminuted fractures. In the same fracture pattern, UBEFs were significantly stiffer than H3-DR (*p* = 0.001) in axial compression and torsion, while only UBEF1 was significantly stiffer than H3-DR in ML bending (*p* = 0.013).

**Conclusions:**

The results demonstrated that the stiffness of the UUEF and UBEF device compares to the reference fixator and may be helpful in maintaining fracture reduction. Fatigue testing and clinical assessment must be conducted to ensure that the objective of bone healing is achievable with such low-cost devices.

## Background

Increasing urbanization and the use of motorcycles in developing countries expose people to high-energy trauma [1]. This is a source of many open lesions of the limbs, particularly in the tibial segment [2]. The generally poor infrastructure and hygiene conditions make it almost impossible to properly treat open fractures using internal osteosynthesis techniques [3]. Thus, the use of external devices provides an opportunity to improve the quality of treatment. There are numerous sophisticated models available on the market, but these are expensive. Many enterprising surgeons have therefore attempted to devise cheaper designs [4]. There is no doubt that these fixators can achieve the same results as those that are more expensive, when used properly [4, 5]. The high costs of commercially available devices present a dilemma to the healthcare industry in poorer countries where there may be patients in need who are unable to afford optimum medical care. One way around this is to reduce the cost of manufacturing a typical fixator so that it is more affordable. This could be brought about by varying the choice of material to make the fixator, the overall product finish, and overall complexity of the design [6]. With all these considerations in mind, the new low-cost external fixators, 304 L stainless steel external fixator (biplanar and unilateral) was specifically designed for the treatment of simple and comminuted patterns. These new designs are intended to provide a biomechanically reliable yet less expensive alternative to currently available devices. The materials used and the tools required are available in almost all developing countries. The construct’s stiffness is its decisive factor, as this ensures correct bone alignment under a mechanical load. When used for fracture management, the stiffness should be sufficient to overcome the forces a patient is subjected to during mobilization to prevent fracture displacement and to avoid nonunion [7]. It is also needed to foster sound callus formation [8]. The aim of this study was to determine the biomechanical characteristics of a low-cost external fixator in comparison with a validated reference fixator.

## Methods

### Bone and fracture model

**Fig. 1:**
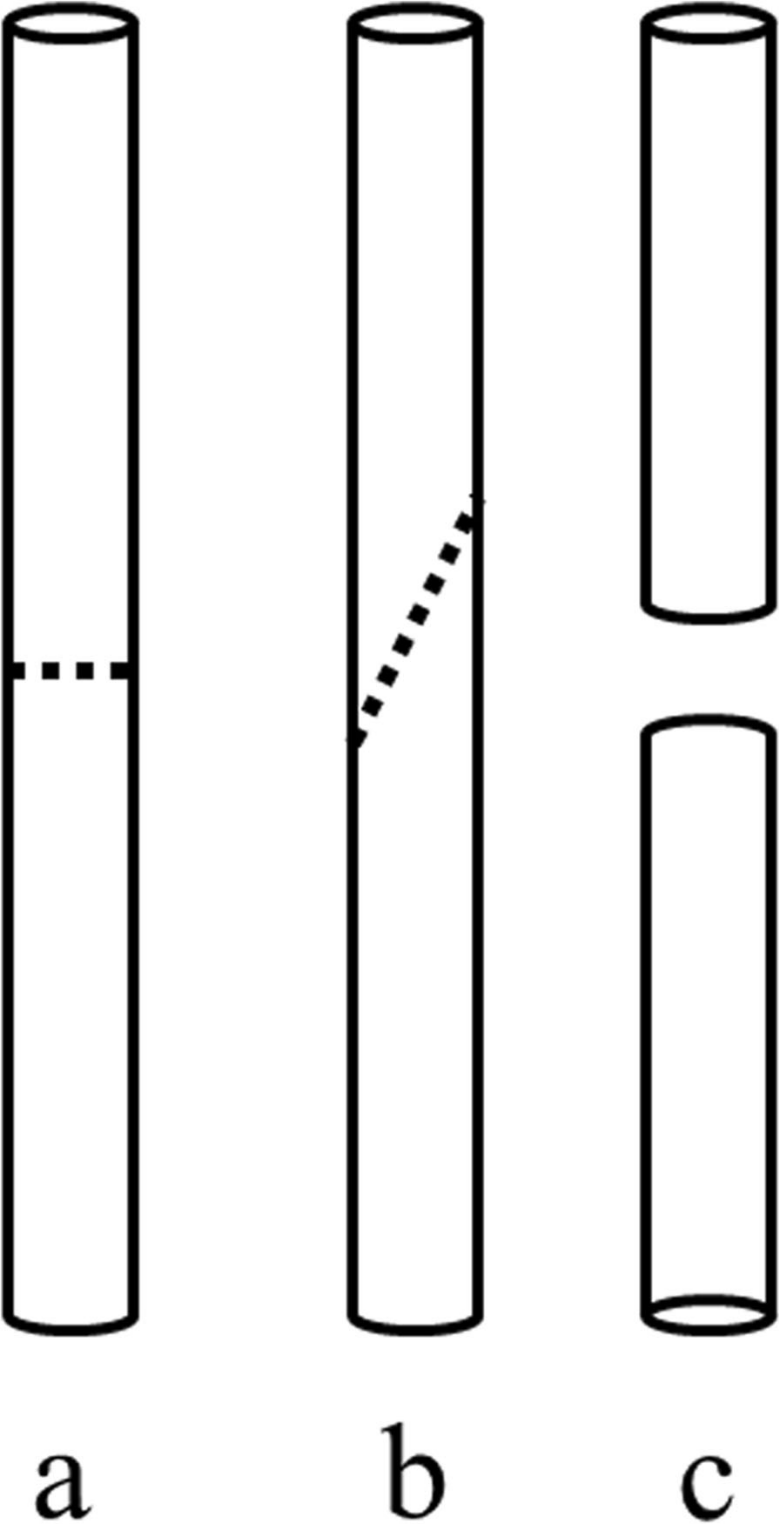
Different types of fractures. **a** Transversal fracture. **b** Oblique fracture. **c** Comminuted fracture

Transverse, oblique, and comminuted fractures (Fig. 1) were created in large-sized, left tibia, synthetic composite bones (model #3402, Sawbones; Pacific Research Laboratories Inc., Vashon, Washington) using a handle saw [9, 10]. The transverse, oblique, and comminuted fractures were set by a unilateral uniplanar external fixator. The biplanar external fixator was only tested in the setting of a comminuted fracture. Twenty-seven constructs were stabilized with uniplanar external fixator (nine constructs each). Nine constructs were stabilized with biplanar external fixator (three constructs each).

### Investigated fixators

**Fig. 2:**
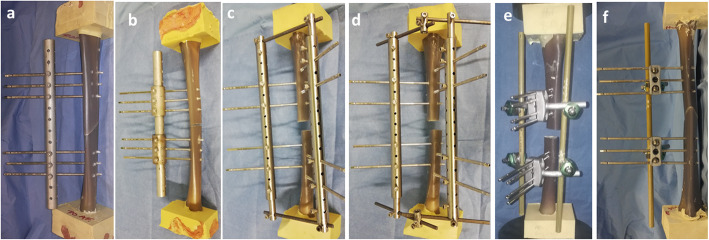
The new fixator design. **a** UUEF1. **b** UUEF2. **c** UBEF1. **d** UBEF2. **e** H3-DR. **f**. H3-SR

The Hoffmann® 3 (H3) fixator (Stryker Trauma AG, Selzach, Switzerland), with a single rod (H3-SR) of 11 mm diameter (Fig. 2 f) and a double rod (H3-DR) of 11 mm diameter [11] (Fig. 2. e), was used as a reference and compared to the low-cost designed external fixators.

**Fig. 3:**
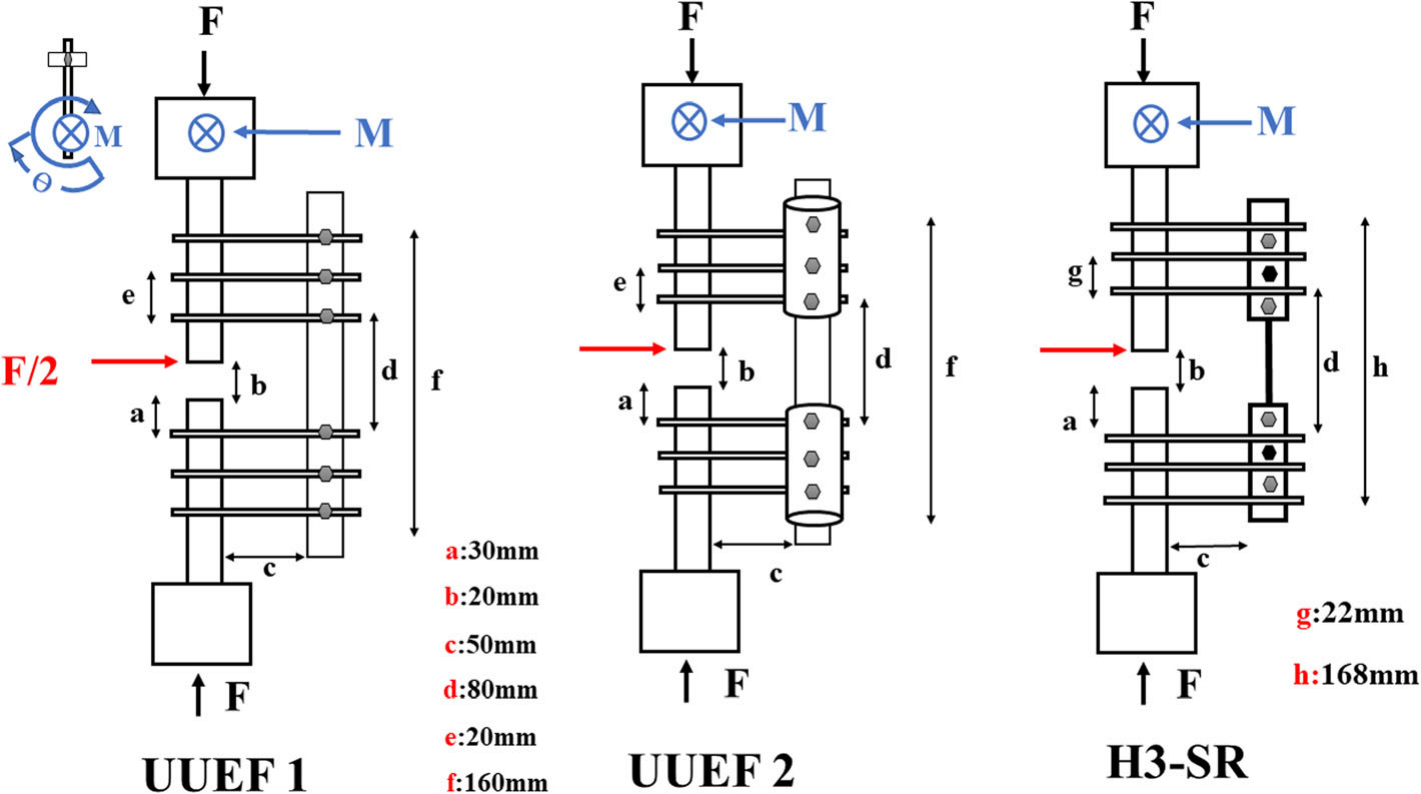
Schematic representations of the external fixator design according to Annex 7 of ASTM F1541-17: F compression force, F/2bending force, M moment

The new fixator design consisted of a unilateral uniplanar external fixator (UUEF1, UUEF2) (Fig. 2 a and b) and unilateral biplanar external fixator (UBEF1, UBEF2) (Fig. 2 c and d). UUEF1 is based on Meyrueis’s fixator (Figs. 2a and 3) [12] and UUEF2 is based on Noor’s fixator (Figs. 2b and 3) [4].

UUEF1 is made of a 304-L stainless steel cylindrical tube. The standard tube has a gauge of 20 mm, a thickness of 3 mm, and a length of 300 mm. The tube is drilled into a perpendicular plane, with holes passing 5.2 mm in diameter, spaced 20 mm apart. The holes accept all types of pins that have a diameter ≤ 5 mm (Fig. 2a).

Threaded holes are perpendicular to those of the pins, which also accept hexagonal and flat-bottom screws that secure the tube/pins.

For UUEF2, the fixation between the pins and the tube connection is ensured by two cylindrical external rings made of stainless steel, with an external diameter of 30 mm, a thickness of 3 mm, and a length of 60 mm. They are composed of three through-holes of 5.2 mm diameter, spaced 20 mm apart for the pins. Five-millimeter diameter thread screws (M5) secure the tube/pins.

**Fig. 4:**
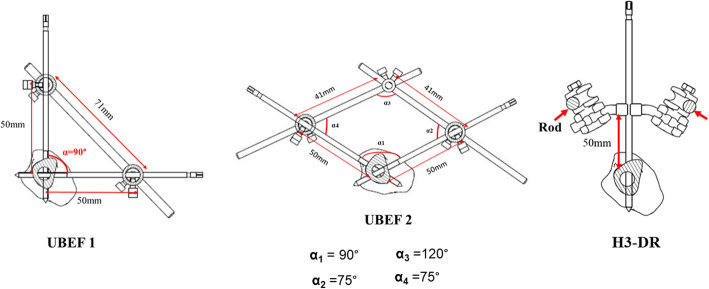
Schematic representation of unilateral biplanar external fixation

UBEF1 is composed of two full bars of 6 mm diameter and 70 mm length, which ensure the connection between the UUEF1 models through four hollow tubes of 13 mm diameter and 40 mm in length that are attached to the four extremities of the hollow tube of UUEF1 (Figs. 2c and 4).

UBEF2 is the same as UBEF1, with two intermediate hollow tubes of 13 mm diameter and 30 mm length, which allow a triangular assembly (Figs. 2d and 4).

### Positioning

Six external fixation frames were tested. Pins of 5 mm in diameter and 180 mm in length, with a 50-mm threaded portion, were used for all tests [6, 8]. Three pins were fixed in each bony fragment for UUEF and H3-SR (Fig. 3), while four pins were used for UBEF and H3-DR. The distance between the bone and rod was 50 mm [11], and the distance between the closest pin from the fracture site was 30 mm. Parameters that were kept constant between the different types of fixators were as follows: (1) diameter of pins, (2) number of pins used in each bony fragment, and (3) distance between bone and longitudinal rod (bone-rod distance) (Figs. 3 and 4).

### Loading modes and test

For mechanical testing, the distal and proximal ends of the sawbones were embedded in molds. All specimens were positioned vertically with a central wood at the bottom of the molds so that the medullary axis fits into the wood. The axis of alignment was controlled with the longitudinal axis of the diaphysis. The molds were filled with polyurethane. The mechanical tests and load conditions were based on the American Society for Testing and Materials (ASTM) standard methods [13].

**Fig. 5:**
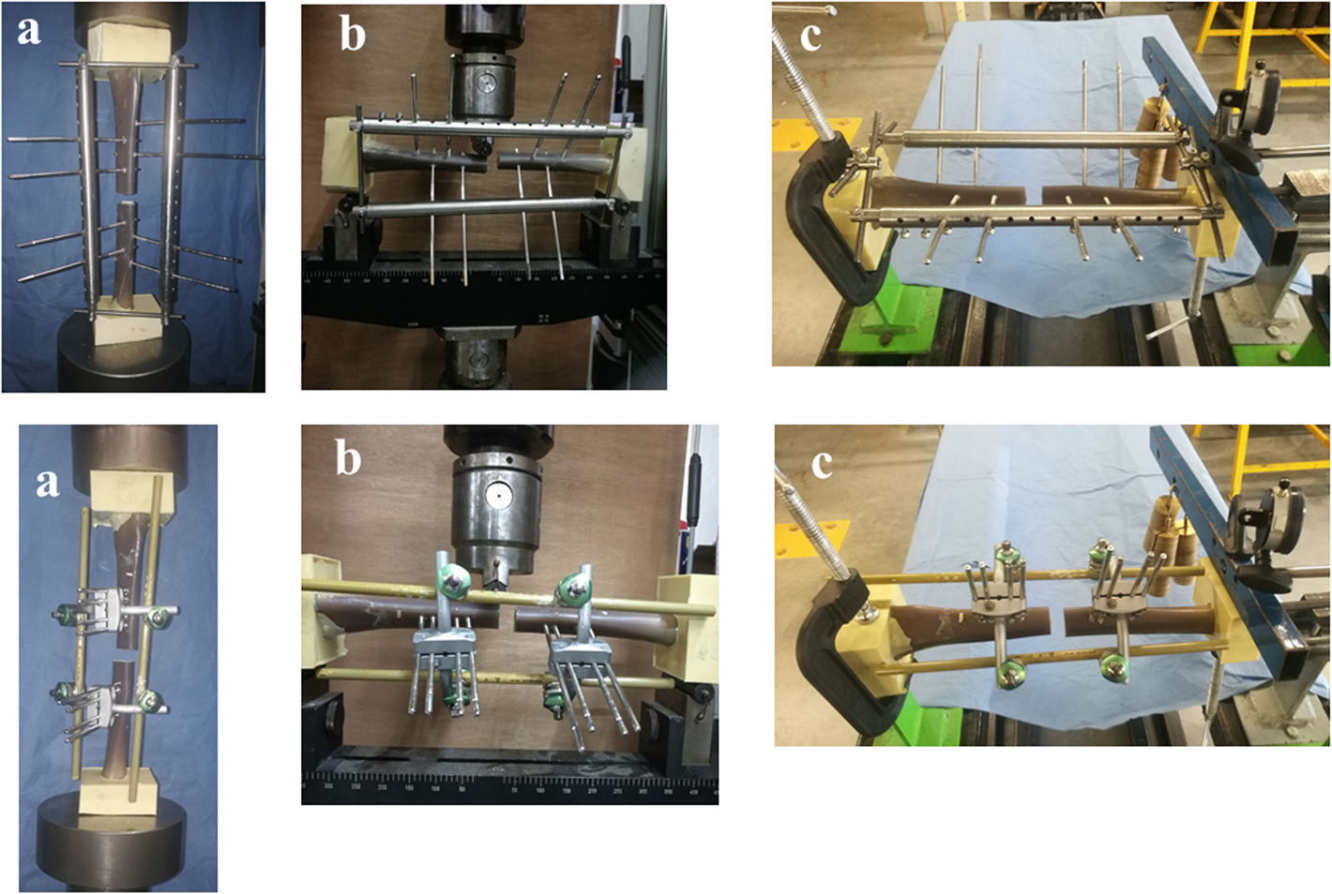
Assembly characteristics and test setup. **a** Compression test. **b** Mediolateral bending test. **c** Torsional test

The stiffness of each fixator was measured in three loading modes: axial compression, mediolateral (ML) bending, and torsion (Fig. 5). The axial compression and AP bending tests were performed using a tension-compression machine (Zwick Roell, type BZ2-MM480xx.EC01, Germany, featuring a maximum 200 kN load cell). Stiffness was computed from the slope of the load-strain curve (N/mm). Each experiment was repeated five times for the three loading modes, after which the averages were computed. For axial compression, each assembly was placed into the machine vertically. Then, a maximum load of 700 N was applied for UUEF and H3-SR, which corresponds to the weight of a 70-kg adult person [14], and a maximum load of 2100 N was applied for UBEF and H3-DR, which corresponds to three times the weight of a 70-kg adult person walking with two crutches. In a pilot study, the static load to failure was determined to be in excess of 2100 N. Hence, an axial load of up to 2100 N was chosen.

In the bending test, the maximum load was such that a maximum deflection of 7 mm for UUEF and 10 mm for UBEF was produced at the fracture site, with a speed of 3 mm min^−1^.

For the torque tests, the proximal part of the bone was clamped, and several static torques were applied to the distal part. The maximal torque allowed was 6 Nm.

A test indicator dial allowed measurement of the angular deflection at each torque. Torsional stiffness was determined as the average slope of the torque-rotation curve and was expressed in Nm degree^−1^.

### Statistics

Statistical analysis was performed using SigmaPlot version 13. We performed a one-way analysis of variance to compare the parametric data (mean ± standard deviation [SD]) of the three fixators’ differences in axial stiffness, ML bending, and torsional stiffness. A Kruskal-Wallis one-way analysis of variance on ranks was used to compare the nonparametric data (median [quartiles]). Post hoc testing was performed using the Tukey test. A level of significance of *p* < 0.05 was used as the threshold for statistical significance.

## Results

Both UUEF models compared favorably to the H3-SR in oblique and transverse fracture patterns (Table 1). UUEF stiffness was equivalent or superior to that of H3-SR.

**Table 1:** ANOVA results of stiffness, expressed as mean ± SD or median [1st–3rd quartiles], after oblique, transversal, and comminuted fractures

Type of configuration	UUEF1	UUEF2	H3-SR	*p* value	UUEF1 vs. UUEF 2	UUEF1 vs. H3-SR	UUEF2 vs. H3-SR
					*p* value	*p* value	*p* value
Oblique fracture							
Axial stiffness N mm^−1^	78.3 ± 5.1	119.7 ± 16.8	67.2 ± 8.1	< 0.001	< 0.001	0.3	< 0.001
ML bending stiffness N mm^−1^	6.2 ± 0.2	8.5 ± 0.7	7.4 ± 0.1	0.004	0.02	0.3	0.3
Torsional stiffness Nm degree^−1^	1.6 ± 0.3	1.5 ± 0.3	0.9 ± 0.2	0.05	0.8	0.06	0.17
Transversal fracture							
Axial stiffness N mm^−1^	1260.1 ± 63.0	1240.1 ± 139.8	1326.2 ± 141.4	0.516	–	–	–
ML bending stiffness N mm^−1^	7.9 ± 0.4	6.0 ± 0.4	5.5 ± 0.9	0.009	0.027	0.009	0.6
Torsional stiffness Nm degree^−1^	1.8 ± 0.3	1.8 ± 0.2	1.4 ± 0.3	0.21	–	–	–
Comminuted fracture							
Axial stiffness N mm^−1^	48.9 ± 3.4	71.8 ± 2.2	35.0 ± 2.1	< 0.001	< 0.001	< 0.001	< 0.001
ML bending stiffness N mm^−1^	4.4 ± 0.0	4.7 ± 0.3	6.5 ± 0.1	< 0.001	0.2	< 0.001	< 0.001
Torsional stiffness Nm degree^−1^	1.8 ± 0.4	1.6 ± 0.2	0.8 ± 0.1	0.016	0.7	0.017	0.042

With regard to comminuted fractures, there was a significant difference between the three fixators (*p* < 0.05) in the three loading modes. A post hoc Tukey test revealed that both UUEFs were stiffer than H3-SR in axial compression and torsion. However, H3-SR was stiffer than both UUEFs in ML bending.

Stiffness in axial compression and torsion for both UBEFs was higher than that for H3-DR (Table 2). UBEF1 was stiffer than H3-DR in ML bending (*p* = 0.013).

**Table 2:** ANOVA results of stiffness, expressed as mean ± SD, after comminuted fractures

Type of configuration	UBEF1	UBEF2	H3-DR	*p* value	UBEF1 vs. UBEF2	UBEF1 vs. H3-DR	UBEF2 vs. H3-DR
					*p* value	*p* value	*p* value
Axial stiffness N mm^−1^	234.7 ± 11.7	228.2 ± 10.2	98.8 ± 4.0	< 0.001	0.53	< 0.001	< 0.001
ML bending stiffness N mm^−1^	62.2 ± 17.1	48.3 ± 15.7	15.2 ± 1.2	0.013	0.4	0.012	0.054
Torsional stiffness Nm degree^−1^	1.5 ± 0.16	1.6 ± 0.04	1.0 ± 0.01	< 0.001	0.8	0.001	< 0.001

There was a significant difference between the three fixators (*p* < 0.05) in the three loading modes. A post hoc Tukey test revealed that the UBEF was stiffer than H3-DR (*p* = 0.001) in axial compression and torsion. UBEF1 was stiffer than H3-DR in ML bending (*p* = 0.013).

## Discussion

Our study was designed to assess the biomechanical properties of UUEF and UBEF frames in a simple and comminuted tibia shaft fracture model. The results indicate that the mechanical behavior of both UUEF and UBEF compared favorably to the reference fixator. UBEF1 stiffness was superior for the comminuted fracture pattern in ML bending.

The treatment of long bone fractures with low-cost external fixators has been reported several times in the literature [4, 6, 15, 16]. Nevertheless, few have been looking into the mechanical properties of their device, prior to clinical use. Goh et al. [6] have previously analyzed the different biomechanical aspects of the simple and low-cost external fixators (AG) compared to the commercially available AO external fixators. The results showed that no significant differences were found in the stiffness of AG and AO fixators under all loading modes. Their mechanical properties appear superior to our uniplanar design but do not significantly differ from our biplanar design.

An external fixation device is also characterized by its simplicity and versatility of application, its ability to minimize soft-tissue damage, its stability at the bone-screw interface [17], its rigidity [18], and its cost-effectiveness [4]. Our frame designs do not need welding as the AG fixator needs. The biplanar frame design offers also more versatility and stability than the AG with pins’ insertion in two planes [17].

However, assessing the overall effective performance of a low-cost external fixator must consider more than just stiffness [6]. In addition to these fundamental requirements [6, 8], the external fixator must be inexpensive [4]. These constructs should also be compatible with patient care and allow the recovery of the soft-tissue envelope [19]. Ideal external fixation systems should be rigid enough to promote fracture healing without secondary loss of reduction, when used as a definitive treatment [8, 20]. Although the Hoffmann® 3 fixator provides excellent versatility [8] and good biomechanical properties [11], its high cost limits its use in developing countries [21].

The development of the callus plays an important role in total fixation system rigidity. Callus with minimal elastic characteristics causes some important variations in the load transmission pattern at the bone-callus-external-fixator structure. A highly rigid external fixator would avoid some micromovements at early consolidation stages but would not prevent load transmission through the callus when this callus appears [22, 23]. However, excessive interfragmentary movement, due to insufficient stiffness of external fixators, can result in deficient callus formation, eventually leading to delayed union or even nonunion with ultimate implant failure [14]. Our external fixators have demonstrated sufficient stiffness. Nevertheless, the correct assessment of callus formation and bone healing has still to be done in an animal fracture model or along a prospective clinical study.

However, this study has limitations. The absence of a soft-tissue envelope, including muscle compartments and the bony pin interface, can influence biomechanical behavior after limb reduction. The influence of the distance between the bone and the rod, as well as the distance between the closest pin and the fracture site, has not been evaluated. These parameters were made constant to primarily evaluate the different construct configurations and their stiffness properties.

For this study, synthetic bone was used instead of cadaver tibias to eliminate variations in geometry. Synthetic bones are considered to have similar structural and mechanical properties as natural bones and thus are close to ideal replicas for standardization in biomechanical analyses [24]. The testing procedure closely followed the ASTM standard methods [13]. This ensured reproducibility and complied with the standard biomechanical testing of external fixators. The force that we applied was around 1% of the maximal force of the 200 kN load cell. The Zwick Roell load cell was tested and calibrated according to ISO 7500-1 standard. For the order of force magnitude we applied, the maximum error for the force was less than 1%. The displacement accuracy was about 2 μm.

Although an increase in stiffness could be provided by increasing the number of pins in any one segment from two to three, the added benefit of increasing the number of pins from three to four is minimal [25]. Shahid et al. [26] reported that using two bars increases the axial compressive stiffness of the fixator by a factor of two. In our model, we observed an increase by three in the comminuted fracture pattern.

Comparison with a reference fixator as the Hoffman 3 was preferred instead of a comparison to another locally developed external fixator or to the Ilizarov design. The objective was to offer similar mechanical properties as what is standardly used in developed countries. Comparison with an Ilizarov system is not appropriate from a mechanical perspective and the use of such device is more complex to handle, partially due to the soft-tissue transfixation, and needs more devices.

UBEF is a system that does not transfixate the anterolateral compartment of the leg, which can achieve very good rigidity [27]. It is a good external fixator system for the treatment of comminuted leg fractures. We postulate that UUEF and UBEF could solve many problems, as they are inexpensive, easy to use, and suitable for both simple and complex fractures. External fixators have been selected as osteosynthesis devices for the treatment of open tibial fractures and certain closed tibial fractures with severe injury to soft tissue [14]. External fixation devices provide a promising and satisfactory alternative for better soft tissue care and for preserving periosteal perfusion to the regions of fracture [10], and they can be implemented both in the provisional and definitive treatment of tibial fractures [9].

All parts of this device, except the pins, can be manufactured in a poor country at a very low cost. They are very cost-effective, except for the Schanz screws. A first economical assessment in Ivory Coast estimates the costs of the frame (without pins) to be the third (9 euros) of a cast immobilization (26 euros) for a tibia fracture.

In less economically developed countries where there is poor healthcare, and many patients are unable to afford optimum medical treatment, such a trade-off may be valuable as a cheaper external fixator that provides simple basic fixation is better than no treatment at all [6]. The frame cost could be also reduced if it was validated to be reusable. Nevertheless, our study does not include fatigue testing and therefore we cannot insure the fatigue properties of our constructs nor its reusability. Indeed, the plastic deformation exhibited by steel at high loads could alter the biomechanics of the fracture site and potentially affect the healing process [28]. Complementary mechanical testing or clinical studies should address this matter.

Medical devices are subject to specific regulations in many (most) countries. The fact that materials and device can be sourced locally and produced does not mean that it will be used without proper clinical assessment and certification in many countries. The frame should also be cleaned, decontaminated, and steam sterilized before use. Some more costs might therefore be expected.

## Conclusions

The low-cost external fixators showed good stiffness properties. They appear suitable for the treatment of both simple and comminuted fractures. They could constitute an alternative to the reference external fixators that are currently sold in the market. However, a fatigue mechanical study and a clinical study are needed to determine their reusability and their ability to promote the bone healing of a fracture.

## Data Availability

The datasets used and/or analyzed during the current study are available from the corresponding author on reasonable request.

## Abbreviations

ASTM: American Society for Testing and Materials
H3-DR: Hoffmann® 3 (H3) fixator with a double rod
H3-SR: Hoffmann® 3 (H3) fixator with a single rod
ML: Mediolateral
SD: Standard deviation
UBEF: Unilateral biplanar external fixator
UUEF: Unilateral uniplanar external fixator
